# Toll‐Like Receptors in the Immunotherapy Era: Dual‐Edged Swords of Tumor Immunity and Clinical Translation

**DOI:** 10.1002/mco2.70308

**Published:** 2025-07-27

**Authors:** Nueraili Maihemuti, Yueli Shi, Kaiyue Zhang, Xinyuan Jiang, Jiahe Chu, Yun Xu, Zhiyong Xu, Kai Wang

**Affiliations:** ^1^ Department of Respiratory and Critical Care Medicine Center for Oncology Medicine the Fourth Affiliated Hospital of School of Medicine, and International School of Medicine, International Institutes of Medicine, Zhejiang University Yiwu China; ^2^ Zhejiang Key Laboratory of Precision Diagnosis and Treatment for Lung Cancer Yiwu China

**Keywords:** cancer, clinical translation, innate immunity, nuclear factor‐κB, Toll‐like receptor

## Abstract

Toll‐like receptors (TLRs), which are critical components of innate immunity, play a significant role in immune responses and deepen our understanding of TLRs. TLRs are a group of transmembrane proteins with similar structures distributed on the cell membrane and endosomes. They trigger downstream acute or chronic inflammatory responses by recognizing different types of pathogen‐associated molecular patterns and damage‐associated molecular patterns. TLRs play pivotal regulatory roles in various tumor types. Over the past few decades, research on TLRs has become increasingly popular, and these molecules can not only directly recognize tumor components as potential targets to activate antitumor immune responses but also act as accomplices to tumor progression and even as driver genes in certain tumor types. Despite their importance, the mechanisms underlying their dual functions remain poorly understood, creating a gap in current research. Here, we summarize the latest advancements in TLR signaling pathways and their application in tumor therapy in recent years, and highlight the development prospects and potential of TLRs in tumor therapy. Moreover, this review underscores the critical regulatory roles of TLRs across various tumor types and explores their prospects in oncology, offering valuable insights for developing targeted therapies and improving cancer outcomes.

## Graphical abstract

This review discussed the signaling pathways of Toll‐like receptors in tumors and their antitumor or protumor regulatory effects in diverse tumors. We also paid attention to the application of Toll‐like receptors in clinical treatment and summarized the clinical trial situation in recent years. Immunotherapy represented by their agonists and antagonists, combined with nanodelivery system and tumor vaccines, is gradually emerging.

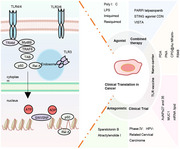

## 1 Introduction

**FIGURE 1:**
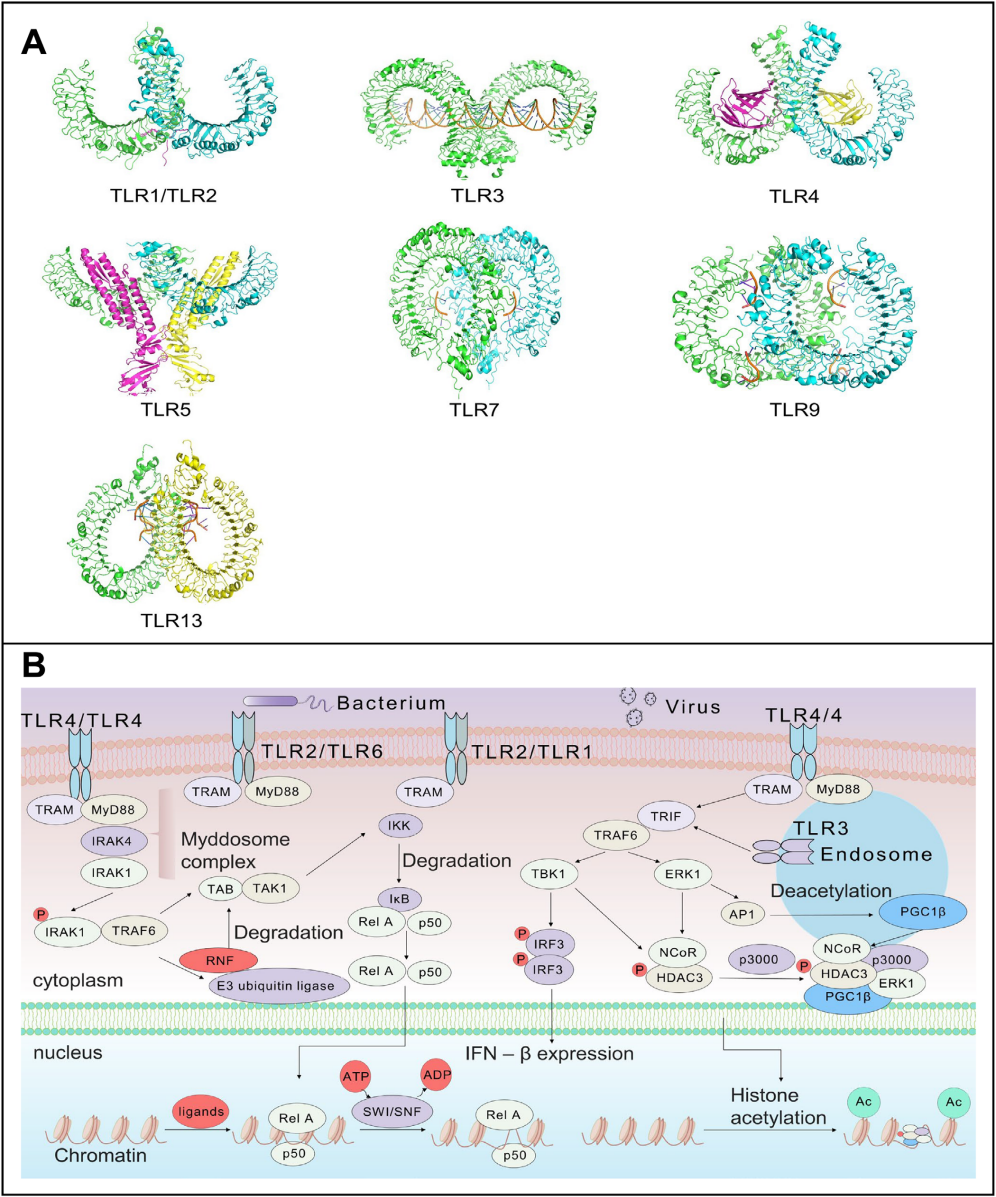
Crystal structure of TLR dimers with ligand complexes and core signaling cascades and functional modules of the TLR pathway. (A) Crystal structure of the TLR3 ectodomain in complex with dsRNA (PDB 3CIF). TLR3 recognizes viral dsRNA, triggering TRIF‐dependent signaling. The dsRNA binds symmetrically along the convex surface of TLR3, inducing dimerization for MyD88‐independent pathway activation. Structural model of the TLR1 (left) and TLR2 (right) heterodimers bound to a triacylated lipopeptide (PDB ID: 2Z7X). This interaction exemplifies the “m”‐shaped dimer architecture characteristic of TLR ligand recognition. Cryo‐EM structure of TLR9 in complex with CpG DNA (PDB ID: 5Y3M). The C‐terminal fragment of TLR9 undergoes pH‐dependent conformational changes in endosomes, exposing the CpG‐binding groove. This interaction triggers MyD88‐dependent signaling, with DNA backbone phosphates coordinating zinc ions in the binding pocket. Crystal structure of murine TLR13 bound to a bacterial 23S ribosomal RNA fragment (PDB ID: 4Z0C). Quaternary structure of the human TLR4‐MD‐2 complex with lipopolysaccharide (LPS) (PDB 3VQ2). MD‐2 acts as a lipid‐binding chaperone, with six acyl chains of LPS embedded in its hydrophobic pocket. This interaction induces TLR4 dimer reorganization, exposing the TIR domain for downstream adaptor recruitment. Crystal structure of the N‐terminal fragment of zebrafish TLR5 in complex with Salmonella flagellin (PDB 3V47). The binding interface involves electrostatic interactions between the TLR5 domain and the flagellin. Notably, zebrafish TLR5 binds a conserved 15‐amino acid epitope, which is distinct from mammalian TLR5 recognition patterns. Cryo‐EM structure of human TLR7 bound to single‐stranded RNA (ssRNA) (PDB ID: 5ZSL). The GU‐rich RNA strand binds within a 25 Å‐long groove of TLR7 LRR8–12, with uridine bases U7 and U15 forming π–π stacking interactions. This pH‐dependent binding induces TLR7 dimerization, activating both the NF‐κB and IRF7 pathways. The structure reveals how TLR7 discriminates viral RNA from host RNA via sequence‐specific base recognition. (B) MyD88‐dependent pathway: Upon ligand recognition, TLRs recruit adaptor protein MyD88, which activates IRAK4/IRAK1 and triggers TRAF6‐mediated K63‐linked ubiquitination. TRAF6 recruits the TAK1–TAB complex, driving IKK phosphorylation and the subsequent degradation of IκB. This releases the NF‐κB dimer (RelA/p50) for nuclear translocation, initiating proinflammatory cytokine gene transcription via chromatin remodeling by SWI/SNF. MyD88‐independent pathway: TLRs activate the TRAM–TRIF complex, which phosphorylates TBK1 to promote IRF3 nuclear translocation and IFN‐β production. TRAF6 synergistically enhances NF‐κB signaling through TAK1 activation via this pathway. TLR, Toll‐like receptor; dsRNA, double‐stranded RNA; MyD88, myeloid differentiation primary response 88; IRAK4/IRAK1, interleukin‐1 receptor‐associated kinase 4/1; TRAF6, TNF receptor‐associated factor 6; TAK1, TGF‐β‐activated kinase 1; TAB, TAK1‐binding protein; IKK, IκB kinase; IκB, inhibitor of κB; NF‐κB, nuclear factor kappa B; RelA/p50, NF‐κB transcription factor subunits; TRAM, TRIF‐related adaptor molecule; TRIF, TIR‐domain‐containing adaptor inducing IFN‐β; TBK1, TANK‐binding kinase 1; IRF3, interferon regulatory factor 3; IFN‐β, interferon‐beta; ERK1, extracellular signal‐regulated kinase 1AP1, activator protein 1; SWI/SNF, SWitch/sucrose nonfermentable; NcoR, nuclear corepressor; HDAC3, histone deacetylase 3, p300, E1A binding protein p300; PGC1β, peroxisome proliferator‐activated receptor gamma coactivator 1‐β.

In 1989, Janeway [1] proposed a hypothesis suggesting that pattern recognition receptors (PRRs) broadly distributed in lymphocytes recognize “pathogen‐associated molecular patterns” (PAMPs), which are responsible for initiating the immune response. This hypothesis was validated in 1996 when Hoffman et al. [2] identified the first PRR, named “Toll,” in Drosophila. Toll‐like receptors (TLRs) are a series of transmembrane sensing receptors that sharing a conserved organismal domain [3, 4], capable of detecting abnormal signals from microbial pathogens and self‐derived damage‐associated molecular patterns (DAMPs) that initiate inflammatory responses upon recognition [5]. TLR family members exhibit a similar structural framework: an N‐terminal LRRNT– leucine‐rich repeat (LRR) module and a C‐terminal LRRCT–LRR module flanking the central hydrophobic LRR domains [6]. The horseshoe‐shaped extracellular region, composed of leucine‐rich fragments repetitive sequence, enables TLRs to efficiently sense PAMPs and DAMPs and mediate robust signaling [7, 8]. Although the TLR family members have similar structures (Figure 1), their specific ligands are diverse and can induce contrasting immune responses after recognition. Based on the current research status, TLR3 is known to be a double‐stranded RNA (dsRNA) [9, 10]; TLR1, 2, and 6 form heterodimers to discriminate a variety of bacterial lipopeptides [11]; TLR5 can be used to identify flagellin [12]; TLR7 and TLR8 can be used to identify single‐stranded RNA (ssRNA) [13, 14, 15]; TLR9 can be used to identify nonmethylated cytosine phosphoguanine (CpG) DNA; and TLR13 can be used to identify 23S ribosomal RNA [16].

Upon recognition of pathogenic or tumor‐derived components by TLRs, naïve myddosomes assemble and dissociate from TLRs on the cell membrane and endosome into the cytoplasm, to mark the initiation of the TLR‐signaling pathway [17]. Formation of the myddosome complex relies on multiple regulatory factors. When the signaling molecule oligomerization process is completed, it activates the downstream nuclear factor‐κB (NF‐κB) signaling pathway, inducing the expression of inflammatory factors and triggering the immune response [18]. During tumorigenesis and progression, distribution of TLRs varies dynamically in tumor cells, infiltrating immune cells, and adjacent tissues. Within the same tissue, distinct TLRs mediate the expression of various inflammatory factors, exerting opposing effects on tumor growth. For instance, in colon cancer (CRC), TLR3 and TLR4 can jointly express chemokines, increase the infiltration of tumor macrophages, and support malignant progression of the tumor [19]. Conversely, injection of TLR agonists can also promote the phagocytic killing effect of antitumor macrophages [20]. This dual functionality will be discussed in detail for different tumor subtypes in subsequent sections.

TLRs represent a cornerstone of innate immunity, playing a pivotal role in pathophysiological of systemic inflammatory responses such as sepsis, tumors, viral infections, and autoimmune disorders. These pathophysiological mechanisms typically involve integrated genetic and proteomic network, with aberrant activation of NF‐κB downstream of TLR serving as a central driver of proinflammatory cascade. In systemic inflammation or cytokine storms, the TLR family, particularly TLR4 and TLR2 activation, becomes markedly upregulated. This is prevalent in infectious diseases. On the one hand, it enhances anti‐infective effect, but excessive activation drives systemic cellular tissue damage caused by inflammation [21, 22]. In systemic sepsis, for instance, TLR4 is persistently activated by invading bacterial communities, driving the secretion of proinflammatory cytokines such as IL‐6 and TNF‐α, whereas epithelial cells suffer loss and apoptosis, compromising host resistance to microorganisms [23, 24]. TLRs also exert protumor effect during the pathophysiological processes of systemic tumors, usually via the specific activation of a TLR subset as a malignant driver gene. A compelling example is tumor lysis syndrome (TLS), a serious complication in rapidly advancing tumors throughout the body. Emerging evidence shows that this systemic pathological condition is associated with elevated extracellular histones, with TLR4‐mediated recognition of these histones emerging as a key mechanism in TLS pathogenesis [25]. During systemic inflammation, especially oxidative damage caused by tumors, phospholipid metabolism reactions produce inflammatory activation products. These products can be recognized by TLR2 receptors, triggering the upregulation of IL‐5 and IL‐13, which in turn triggers cell apoptosis, increases the expression of proinflammatory cytokines, enhances anti‐infective effects, and promotes systemic pathological changes such as tumor proliferation and invasion [26].

In cancer treatment, immunotherapy, harnessing the immune system to recognize and eliminate tumors, has become a pillar of tumor treatment [27]. TLRs are ubiquitously distributed in tumors and immune cells. A total of 11,057 tissues were identified in the Cancer Genome Atlas. Compared with other TLR genes, TLR9 is almost unexpressed, resulting in a lower correlation between TLR9 and immune estimation scores and matrix estimation scores [28]. All TLRs exhibit differential associations with tumor immune subtypes, demonstrating distinct distribution profiles across immune classification models [28]. This dual functionality of TLRs—acting as tumor driver genes to promote growth/invasion while activating downstream pathways to inhibit tumor progression and induce apoptosis—positions specific TLRs as promising therapeutic targets. It suggests that their agonists and antagonists can served as immunotherapies or adjuvants, or that they can drive immune cells to exert killing effects through downstream signaling molecules. Although TLRs hold great potential for cancer treatment and have been widely studied for their role in targeted immunotherapy, their full value remains to be elucidated. In this review, we focus on the different roles of the TLRs family in signal transduction and tumor immune response, summarizing their positive or negative regulatory roles in certain tumor types and introducing treatment strategies based on these differential regulatory mechanisms in recent years.

## 2 Classification and Overview of TLR Signaling Pathways

The TLR family comprises multiple members with nonidentical downstream signaling pathways, yet they exhibit conserved features and share similar key mediators. All TLR signaling cascades converge on the activation of immune response‐related gene expression (Figure 1). Upon recognition of a PAMP or DAMP, TLRs initiate specific intracellular signaling events that trigger transcription factor (TF) activation, causing TF activation and nuclear translocation, which regulates the expression of immune‐related genes such as inflammatory cytokines and antimicrobial peptides. Notably, TLR receptor intracellular signaling pathways that have been categorized into two distinct forms: MyD88 (myeloid differentiation factor 88)‐dependent signaling pathways and MyD88‐independent signaling pathways [29].

### 2.1 Mechanisms of the MyD88‐Dependent Pathway

MyD88 serves as a crucial adaptor in most TLR signaling cascades. In the MyD88‐dependent signaling pathway, activated TLRs recruit MyD88, which engages IRAK4 (interleukin‐1 [IL‐1] receptor‐associated kinase 4) through homotypic alteration via its death domain. This interaction facilitates later IRAK1 (IL‐1 receptor‐associated kinase 1) translocation and phosphorylation [30, 31]. The IRAK family orchestrates diverse signaling processes, including the tumor inflammatory response, proliferation, and invasion. IRAK4 and IRAK1 are active kinases. Phosphorylated IRAK1 is dissociates from the complex and binds to tumor necrosis factor receptor (TNFR)‐associated receptor (TRAF6), triggering activation of TAK‐1/TAB (TGF β‐activated kinase/TAK1 binding protein) [32, 33]. The latter increases the degree of reactivity of the IκB kinase (IKK) complex, promoting its phosphorylation, which results in its degradation, in combination with nuclear factor kappa B (IκB) inhibitors. Consequently, the formation of NF‐κB dimers, comprising p65 (RelA), c‐Rel, p50, and IκB, is disrupted, enabling their nuclear translocation and enhancing the transcription of inflammatory genes, including tumor necrosis factor‐alpha (TNF‐α), IL‐1β, IL‐6, and IL‐12 [34]. TRAF6 acts as a crucial intermediate node in the signaling pathways [35]. TRAF6 consists of an N‐terminal zinc finger domain and C‐terminal TRAF domain. The former governs E3 ubiquitin ligase activity, whereas the latter catalyzes K63‐linked ubiquitination, a critical mechanism for TAK1 activation and NF‐κB signal transduction [36, 37].

### 2.2 Mechanisms of the MyD88‐Nondependent Pathway

MyD88‐independent activation of the TLR pathway, which is mainly surveyed during the activation of TLR3 and TLR4, has been extensively investigated. TLR3 directly engages the TIR domain adaptor protein (TRIF) [38], whereas other TLRs indirectly recruit TRIF by utilizing TRIF‐associated adaptor molecules (TRAMs) as mediators [39]. The formation of the signaling complex between TRIF and TRAF6 activates TBK1, leading to phosphorylation of interferon (IFN) regulatory factor 3 (IRF3). This phosphorylation event promotes dimerization of IRF3, leading to its translocation into the nucleus and enhanced transcription of IFN‐β. IFN‐β stimulates the transcription of IFN‐stimulated genes (ISGs) via the Janus kinase signaling pathway and signal transducer and activator of transcription (STAT) pathways [40, 41]. The MyD88‐nondependent pathway activates the IFN‐β/STAT1 cascade through P38 mitogen‐activated protein kinase (MAPK) and c‐Jun N‐terminal kinase, which are critical for IFN‐γ‐induced production of C‐X‐C motif chemokine ligand 10 (CXCL10) [42].

### 2.3 Effects of Histone‐Mediated Chromatin Remodeling on the Transcriptional Regulation of Downstream Target Genes via TLR Signaling

Chromatin accessibility profoundly influences gene expression, with heterochromatin remaining transcriptionally repressed and euchromatin permitting access to transcriptional machinery upon TF nuclear translocation [43, 44]. Highly compacted heterochromatin c resists engagement by transcriptional mechanisms, whereas uncompacted euchromatin can. Hence, it is feasible to regulate gene expression after TF enter the nucleus. The switch/sucrose unfermented (SWI/SNF) complex regulates chromatin accessibility through ATP‐dependent nucleosome sliding and expulsion. Upon stimulation, the downstream activated SRTF motifs of TLRs, AP‐1, NF‐κB, and the IRF family, accumulate in the incorporable domain of SWI/SNF [45, 46]. In the ATAC‐seq and single‐cell ATAC‐seq analyses, the absence of the NF‐κB subunits RelA and c‐Rel led to the loss of high‐confidence ATAC‐seq peak remodeling, and CRISPR–Cas9 mutations in the NF‐κB‐binding motif disrupted the remodeling. By collaborating with other inducers, including IRF3 and MAP, the defined region is endowed with remodeling selectivity. Notably, NF‐κB plays a most critical role in the signaling pathway activated by TLRs because of its unique contribution in promoting nucleosome remodeling and stabilizing enhancers and promoters assembled into euchromatin [47]. A recent study has demonstrated that the SWI/SNF complex facilitates the assembly of compacted chromatin via local TF pathways in vitro, which in turn stabilizes the combination of pioneer factors (PU.1) [48, 49]. Additionally, the complex formed by the nuclear receptor corepressor (NCOR) and histone deacetylase 3 (HDAC3) regulates inflammatory gene expression through NF‐κB signaling, which can be achieved by TLR4 recruiting the complex or by deacetylating modified histones to inhibit their transcriptional function [50].

### 2.4 Interaction Between TLR Signaling and Other Signaling Pathways

Similarly, other signaling molecules have a crucial impact on the TLR‐guided pathway. Upon activation, the TLR–NF‐κB signaling cascade upregulates FRZB expression. Notably, FRZB functions as an endogenous antagonist of the canonical Wnt signaling pathway, thereby inhibiting Wnt/β‐catenin signaling [51]. The signal lymphocyte activation molecule family 7 (SLAMF7) collaborates with Src homology 2 domain‐containing inositol‐5‐phosphatase 1 to downregulate the activation of MAPK and NF‐κB downstream of TLRs, which is achieved by inhibiting TRAF6 [52]. SLAMF7 knockdown significantly reduces the mRNA expression of TLR‐induced proinflammatory markers [53]. The AHR–TLR signaling pathway is involved in regulating MyD88 expression. Upon binding to the corresponding ligand, the former mediates STAT3 through the latter; however, a single protein level change cannot independently regulate STAT3 [54]. PSMC5 is involved in recognizing marker proteins in the ubiquitination degradation pathway. Lipopolysaccharide (LPS)–TLR4 stimulation reveals that silencing PSMC5 inhibits the secretion of TLR4‐induced inflammatory factors and reduces the activation level of p65, demonstrating the involvement of PSMC5 in TLR4–MyD88‐dependent p65 activation [55]. TIRAP (or Mal) acts as an intracellular TLR signaling mediator, and patients with TIRAP polymorphisms (rs3802814 and rs8177374) exhibit significantly shorter progression‐free survival (PFS) and overall survival (*p* < 0.05) [56]. IRF1 blocks the IFN pathway mediated by TLRs, thereby reducing the cytotoxicity effect of effector T and NK cells on tumor cells, which is accomplished by intercepting the ISGs [57].

Crosstalk exists between TLRs and other intracellular receptor pathways. TLR signaling typically enhances and prolongs STING‐mediated immune responses via NF‐κB. The degradation of STING relies on the transport of microtubules to lysosomes, whereas the activation of TLRs/NF‐κB inhibits this transport process, thereby delaying the degradation of STING and enhancing IFN‐I and inflammatory responses [58]. The FAS receptor shares chaperone CD14 with TLRs and activates downstream TRIF. Therefore, FAS signaling depends on the binding of CD14 to TLRs [59]. The IFN signaling pathway, mediated exclusively by TLR3 and TLR4, relies on endoplasmic reticulum‐associated TRIF to activate downstream TNFRs [60].

### 2.5 Specific Functions of TLR Subgroups

Members of the TLRs family share the common signaling molecules for transmitting signals initiated by the interaction between TLRs and ligands; yet their specific functions diverge profoundly. Building on the previous section's discussion of ligand‐mediated chromatin accessibility regulation [61]. Here, we summarize the discrepancies in signaling between different TLR subgroups and their impact on immune responses. Research on cDC has found that activation of TLR2/TLR1 heterodimers rapidly polarizes the inflammatory milieu into a proinflammatory state. Proteomic analyses reveal that TLR activation reshapes cDC surface markers, with notable downregulation of inhibitory molecules like PD‐1 and CD22 [62]. In a study targeting mammalian DC, TLR2, TLR4, and TLR5 exhibited the same immune activating effect when faced with their specific ligands, driving a significant upregulation of IL‐1 β, IL‐6, and IL‐12p40 compared with the control group [63]. Research on TLR2‐deficient macrophages has shown that TLR2 knockout leads to a decrease in prostaglandin PGD2 secretion, attenuating the macrophage‐driven inflammatory response [64]. TLR4 is the most extensively studied subgroup within the TLR family, and its specific ligand, LPS, is widely used in immune activation assay [65]. TLR4 regulates the inflammatory response by secreting cytokines and chemokines, such as TNF‐α, IL‐1 β, IL‐6, promoting macrophage differentiation toward the M1 type and promote systemic inflammation [66, 67, 68, 69]. TLR3 increases the secretion and activation of TNF‐α, IL‐1, IL‐6, and IFN‐I by recognizing dsRNA, thereby promoting an antitumor immune response [70]. TLR7 and TLR8 have similar roles in activating immune responses and share the same ligand [71]. Upon activation, both can secrete proinflammatory factors such as IL‐1, IL‐6, and TNF‐α, as well as anti‐inflammatory factors, including IL‐10, balancing immune homeostasis [72]. TLR9 also exhibits a dual regulatory effect on inflammation and can inhibit inflammation via the IFN signaling pathway [73, 74]. TLR9–IL1 signaling initiates immune responses induced by CD8+T cells [75] and can also promote macrophage polarization into M1 type through IFN signaling to exert antitumor effects [76]. Activation of TLR9–MyD88–STAT3 signaling suppresses the progression of inflammatory responses [77].

### 2.6 Signal Interaction Between TLRs and Immune Checkpoint Molecules

Immune checkpoint molecules such as PD‐1/PD‐L1, cytotoxic T lymphocyte‐associated protein 4 (CTLA‐4), and lymphocyte activation gene 3 are critical for tumor immune evasion, and emerging evidence highlights signaling crosstalk between TLRs and these inhibitory pathways [78]. Activation of TLR directly induces the transcription of PD‐L1 by NF‐κB or indirectly increases the expression level of PD‐L1 by upregulating the PD‐L1 promoter through IFN signaling [79]. The relationship between TLR4 and PD‐L1 was studied using immunohistochemistry and gene enrichment analysis, and it was found that TLR4 can increase the expression of PD‐L1 [80]. The upregulation of PD‐L1 by TLR4 is achieved through the activation of MAPK signaling. Additionally, TLR4‐mediated IL‐10 production has been proposed as an alternative regulatory axis for PD‐L1 induction [81, 82]. The upregulation of PD‐L1 expression mediated by TLR–IL‐10 was confirmed by histopathological analysis in a mouse model of sepsis [83]. TLR9 promotes the expression of PD‐L1 by regulating STAT3 signaling and enabling tumor cells to escape immune surveillance. Reducing TLR9 expression effectively sensitizes tumor cells to targeted PD‐L1 therapy [84, 85]. In addition to the inhibitory effect of TLR–STAT3, when the TLR–IFN signaling pathway is activated, the IRF1 TF can activate the expression of STAT1, thereby upregulating the expression of PD‐L1 and dampening the antitumor inflammatory response [57, 86]. The signal interaction between TLRs and inhibitory markers may provide a potential direction for sensitizing immunotherapy.

## 3 TLRs in Tumor Malignancy

### 3.1 The Polymorphism Characteristics of TLRs in Tumors

Distinct members of the TLR family have unique physiological structures that underlie various immune responses [87, 88]. Single nucleotide polymorphism (SNP) characteristics of TLRs play a pivotal role in mediating interindividual differences in immune responses [89, 90, 91]. While soluble mediators, such as ILs and chemokines, are characteristic molecules of TLR‐mediated inflammatory responses [92], whereas SNP are crucial interfering factors [93, 94]. Dysregulated immune response often stems from aberrant state of the downstream signaling molecules involved in TLR signaling. For instance, ATG16L1^T300A^ variation hyperactivates the TLR–NF‐κB signaling pathway by enhancing ubiquitination of TRAF6, and ultimately leads to exaggerated cytokine expression [95]. Moreover, SNP rs3761624 in TLR8 gene can interact with the widely studied tumor suppressor gene p53 in tumors, upregulating TLR8 protein transcription and IL‐6 cytokine expression [96]. This p53–SNP‐dependent p53–TLR axis is also observed in TLR3 and TLR5 [97, 98]. We have summarized research on SNP induced TLR‐signaling discrepancies in tumors over the past decade.

A study on the frequency of TLR polymorphisms found that the frequency of the TLR4 rs4986790 SNP was significantly elevated in patients with ovarian cancer, which may indicate its association with the development of ovarian cancer. Further verification is required to determine this specific situation [99]. In a study of TLR polymorphisms in oral squamous cell carcinoma (OSCC), molecular analyses were conducted on four SNPs of TLR4 and TLR9, and the results revealed that these polymorphic features may be associated with an increased cancer susceptibility [100]. Through the study of hepatocellular carcinoma (HCC) patients caused by hepatitis B virus (HBV), TLR3 and TLR4 polymorphisms served as biomarkers of virus‐related tumors [101]. The polymorphisms rs1640827 and rs17163737 of TLR5 enhance Helicobacter pylori susceptibility, elevating the risk of gastric cancer (GC) [102]. Currently, SNP research of TLR predominantly focused on pathogenic microorganism infectious diseases and autoimmune diseases. For oncology, while polymorphic associations have been identified, mechanistic insights and clinical translation remain underexplored.

### 3.2 The Impact of Gut Microbiota on TLRs in Tumor Background

The human intestine harbors large and diverse gut microbiota, comprising symbiotic bacteria, viruses, fungi, and other microorganisms have the potential to manipulate TLR‐mediated immune responses by presenting the binding of PAMPs to and activating TLRs through their components [103, 104]. Invasive microbial infections disrupt the homeostasis of the gut microbiota, directly activating TLR signaling and triggering a large accumulation of proinflammatory signaling molecules. This contrasts with invasive and commensal microorganisms, which are normally tolerated by gut TLRs [105, 106]. In the context of tumors, commensal and invasive microorganisms promote or inhibit tumors through TLR‐mediated immune modulation, inducing cytotoxic immune cell infiltration, tumor‐associated macrophage (TAM) polarization, and other mechanisms, which can serve as adjuvants for immunotherapy [107]. Dysbiosis of microbiota is prevalent in tumors. Similar to the interaction with the tumor microenvironment, tumors and gut microbiota also interact with each other. For example, *Helicobacter pylori* infection activates multiple TLRs, including TLR4 and TLR5, driving protumor inflammatory response. The polymorphism of TLRs in tumors, in turn, promotes the development of gut microbiota toward the tumor microbiota [108, 109]. Research on the bidirectional interaction between the gut microbiota and tumors is going momentum, and TLR, as receptors that recognize both microorganisms and tumors simultaneously, holds promise for targeted gut microbiota therapy for tumors.

### 3.3 Microbiota‐Driven Carcinogenesis

**FIGURE 2:**
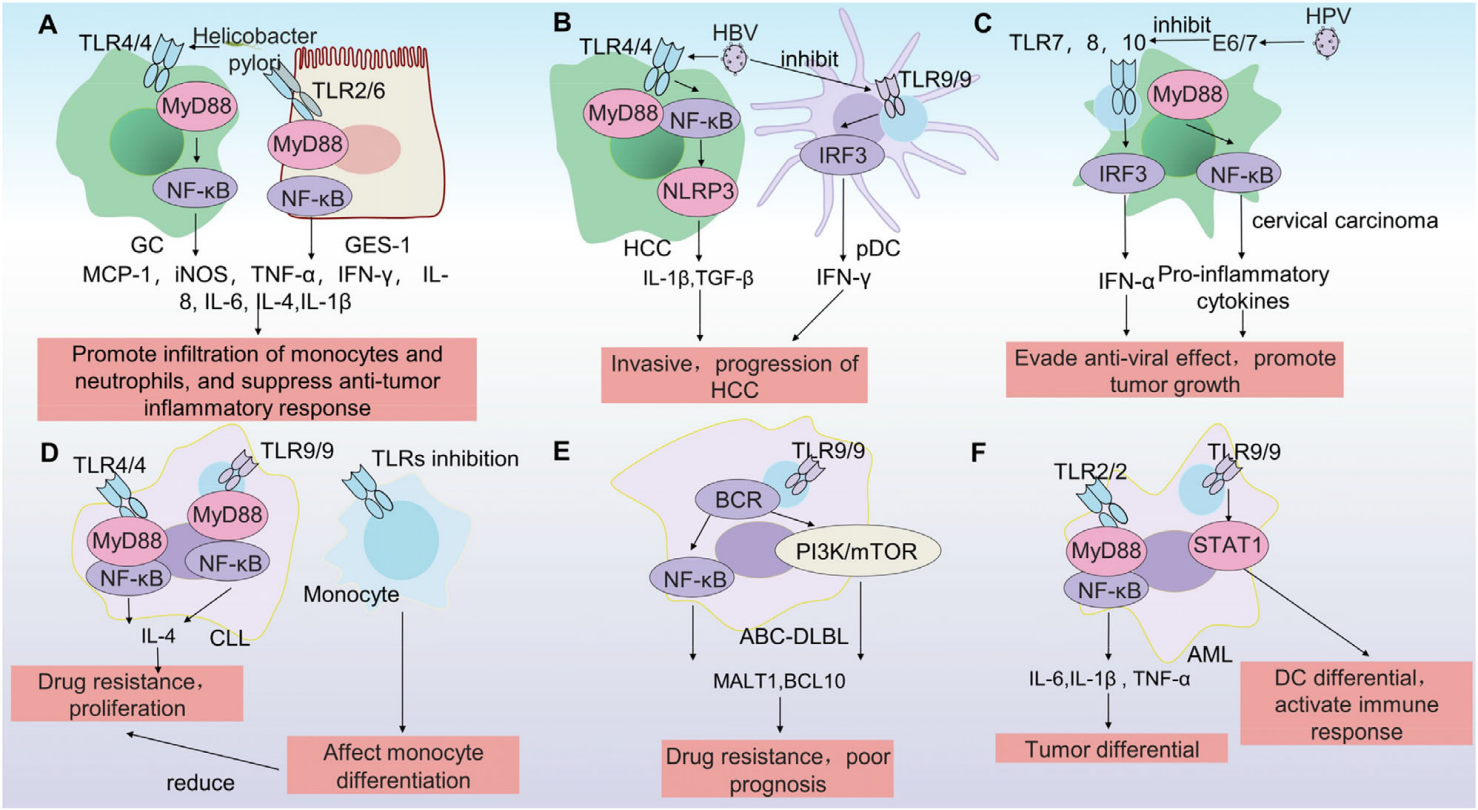
Features of TLRs in microbial‐related tumors and lymphatic hematopoietic system tumors. (A) In *Helicobacter pylori*‐associated gastric cancer, TLR4 activation on tumor cells and TLR2/6 heterodimer activation on gastric mucosal epithelial cells synergistically induce cytokine release, promoting monocyte/neutrophil infiltration while suppressing antitumor immune responses. (B) In HBV‐related hepatocellular carcinoma, dual activation of the TLR4‐mediated NF‐κB‒NLRP3 axis in tumor cells and TLR9 suppression in pDCs create a tumor‐promoting microenvironment through impaired IFN‐γ production and increased cancer invasiveness. (C) In HPV‐driven cervical cancer, E6/E7‐mediated suppression of tumor cell surface TLR7/8/9 receptors enable immune evasion by blocking antiviral signaling pathways, thereby accelerating tumor progression. (D) In chronic lymphocytic leukemia, TLR9/NF‐κB hyperactivation in malignant B cells drives chemoresistance and disease progression, whereas TLR inhibition in infiltrating monocytes disrupts their differentiation‐mediated antitumor functions. (E) In diffuse large B‐cell lymphoma, constitutive TLR9 signaling via the PI3K–mTOR and NF‐κB dual pathways not only mediates therapeutic resistance but is also is correlated with adverse clinical outcomes. (F) In acute monocytic leukemia, divergent TLR2–NF‐κB (tumor differentiation) and TLR9–STAT1 (DC‐mediated immunity) activation orchestrate a complex interplay between leukemogenesis and host immune surveillance. TLR, Toll‐like receptor; DC, dendritic cells; NF‐κB, nuclear factor kappa B; NLRP3, nucleotide‐binding domain and leucine‐rich repeat containing protein 3; IFN‐γ, interferon‐γ; HPV, human papillomavirus; PI3K, phosphatidylinositol 3‐kinase; mTOR, mechanistic target of rapamycin; STAT, signal transducer and activator of transcription.

The immune response mediated by TLRs intersects with tumor progression, including the direct activation of downstream TLR signals by tumor‐derived components, as well as the detection of tumor‐associated pathogens (Figure 2). Chronic inflammation triggered by pathogens like *Helicobacter pylori* and Epstein–Barr virus (EBV) [110], which are important pathogenic factors for GC [111, 112, 113, 114]. For instance, 16S rRNA profiling reveals association with TLR4, *B. fragilis*, and TLR4 in the peripheral blood of patients with CRC [115]. Microbial infections are also implicated in HCC [116, 117] and OSCC [100]. The components of pathogenic microorganisms activate TLRs as PAMPs and driving distinct inflammatory landscapes [118].

As the third leading cause of cancer mortality rate, HCC has a poor prognosis and is susceptible to drug resistance, necessitating it urgent to search for novel targets for HCC [119]. A consensus link HCC to HBV infection [120]. TLRs distributed in HCC cells and adjacent tissues play important roles in tumor virus recognition, and the NF‐κB pathway can inhibit microbe replication and tumor progression [121, 122]. Downregulation of TLR3, TLR7, and TLR9 emerge as a potent risk factor for HCC. During tumor progression, microbes continually suppress TLR signaling pathways. For example, HBsAg inhibits IFN signaling by downregulating the transcription of the TLR9 gene in infiltrated pDCs [123]. By blocking IRF‐3, NF‐κB, MAPK, and ERK 1/2, HBsAg, HBeAg, and HBV viral particles, among other HBV components, eliminate TLR‐mediated antimicrobial activity in hepatic sinusoidal endothelial cells (LSECs) [120]. Some TLR signals also contribute to tumor progression, and HBsAg can increase the invasiveness of HBV‐induced HCC cells by enhancing TLR2 transcription [124]. Some microorganisms, such as Streptococcus maltophilus, which promote the senescence‐associated secretory phenotype by utilizing activated TLR4‒NF‐κB signaling in liver cells, are distinct from HBV. The latter can promote the assembly of NLRP3 (nucleotide‐binding domain and leucine‐rich repeat containing protein 3) inflammatory complexes, and downstream inflammatory factors contribute to HCC progression [116].

Among the common cervical cancers in women worldwide, human papillomavirus (HPV) infection is the most significant factor, and HPV 16 and 18 evade antiviral effects by reducing downstream activation of the NF‐κB and IFN signaling pathways of TLRs through the E6/7 protein and promoting tumor development [125]. The transcription of TLR7, TLR8, and TLR10 is negatively regulated in HPV‐induced cervical cancer [126]. The HPV16 E7 oncogene can downregulate the expression of TLR9, which mainly senses HPV RNA. TLR7 expression is a predictive factor for HPV clearance in women with cervical HPV infections [127]. TLR2, TLR3, TLR7, TLR8, and TLR9 play critical roles in clearing HPV16 and can be downregulated to varying degrees. However, TLR4 is upregulated in cervical cancer cells and associated with poor lymph node metastasis [128].

GC high mortality and drug resistance highlight unmet clinical needs [129]. Compared with normal individuals, infiltrating DCs and monocytes in GC patients overexpress TLR2, TLR3, TLR4, and TLR9, which is positively correlating with GC progression [130]. Bioinformatics analysis of key miRNAs related to TLR activation in GC cells revealed that miR‐16‐5p governs TLR activation in GC cells [131]. Variants of TLR4, TLR5, and TLR9 have been identified as potentially threatening elements of GC [132]. *Helicobacter pylori* is a persistent pathogen that stimulates NF‐κB activity and promotes monocyte infiltration by upregulating monocyte chemoattractant protein‐1. Its components activate TLR4 to upregulate the expression of inflammatory factors, including inducible nitric oxide synthase (iNOS), TNF‐α, IFN‐γ, IL‐8, IL‐6, IL‐4, and IL‐1β [105]. The expression of TLR6 first increased and then decreased during treatment with *Helicobacter pylori*. Restoring their levels can increase the expression of IL‐1β and IL‐8 in GES‐1 (gastric mucosal epithelial) cells, thereby increasing neutrophil infiltration and exerting an inhibitory effect on *H. pylori*. Long‐term *Helicobacter pylori* infection can lead to a decrease in TLR6 sensitivity and downregulation of inflammatory cytokine expression through TLR6/JNK signaling [133]. In EBV‐induced GC, TLR9 signaling is closely related to disease progression. EBV+ patients have significantly increased CD4+ and TLR‐9+ T lymphocytes and CD19+ and TLR‐9+ B lymphocytes, whereas increased TLR9 expression is associated with a poor prognosis [111, 134].

### 3.4 Hematolymphoid Tumors

The TLR signaling pathway is ubiquitously distributed in immune cells and exerts an enormous influence on mediating immune responses orchestration and immune cell functionality (Figure 2). The functions of TLRs in lymphatic hematopoietic system tumors also differ. Aberrant activation of specific TLRs drives tumor progression, while inhibition of TLRs involved in immune cell differentiation/activation impairs antitumor immunity [135].

#### 3.4.1 Acute Myeloid Leukemia

Acute myeloid leukemia (AML) is a prevalent hematological malignancy, comprising a significant account for a certain proportion of leukemias, and is characterized mainly by hematopoietic stem cell differentiation arrest. Recent studies have highlighted pivotal roles for TLR signaling pathways in AML pathogenesis.

In AML, TLR activation upregulates glutathione peroxidase 1 (GPX1) expression. Elevated GPX1 levels are correlating with poor prognosis and drug resistance in AML patients [136]. A hallmark of AML is stasis of hematopoietic stem cell differentiation. Intriguingly, stimulation of the TLR2 pathway has been shown to directly induce differentiation of AML cells [137]. TLR–STAT1 activation in AML cells mediates DC differentiation and orchestrates immune response [138].

In an investigation of three TLR9 polymorphisms, rs187084, rs5743836, and rs352140 were found to be associated with an increased susceptibility of developing AML [139]. ECDD–S16 inhibits pyroptosis in Raw264.7 (mouse monocyte macrophage leukemia cells) cells by activating TLR ligands. The decline in focal apoptosis observed in ECDD–S16 cells is mechanistically linked to defective intracellular acidification, which also decreases reactive oxygen levels [140].

#### 3.4.2 Chronic Lymphocytic Leukemia

Systemic expansion of monoclonal B lymphocytosis characteristically manifests as chronic lymphocytic leukemia (CLL). It is prevalent in middle‐aged and elderly individuals [141, 142]. Aberrant TLR/NF‐κB signaling facilitates CLL proliferation and progression, potentially by modulating resistance to apoptosis and microenvironmental interactions. TLR receptors distributed on CLL cells are capable of mediating drug resistance and antiapoptotic effects through downstream TNF and IFN signaling [142, 143]. In leukemia patients, signals within the tumor microenvironment activate TLR/NF‐κB signaling, thereby preventing apoptosis [142]. The TLR signaling pathway promotes the proliferation of CLL cells, with TLR9 enriched in CLL. Inhibition of TLR7/9 signaling disrupts monocyte‐to‐macrophage differentiation, thereby suppressing CLL cell proliferation [144]. TLR4 is associated with proliferation and drug resistance of CLL cells [145]. Consistent with previous findings, a recent study revealed a dynamic reciprocity between CLL and its tumor microenvironment, where mutual TLR signaling activation enables evasion of apoptosis [146].

#### 3.4.3 Lymphoma

Activation of the TLR pathway has been implicated in rare lymphoma complications [147]. In activated B‐cell‐like diffuse large B‐cell lymphoma (ABC‐DLBCL) cells, TLR9 triggers assembly of the My‐T–BCR multiprotein complex, which potently activates the MyD88 pathway. This activation triggers aberrant BCR‐dependent signaling through the NF‐κB axis, thereby sustaining malignant survival and proliferation [148, 149]. This BCR‐dependent activation of TLR9 can also be abnormally amplified by the lymphoid TME, conferring resistance to compounds targeting the BCR–TLR9 pathway members, such as Bruton tyrosine kinase and mucosa‐associated lymphoid tissue lymphoma translocation protein 1 (MALT1) [150].

### 3.5 Solid Tumors

**FIGURE 3:**
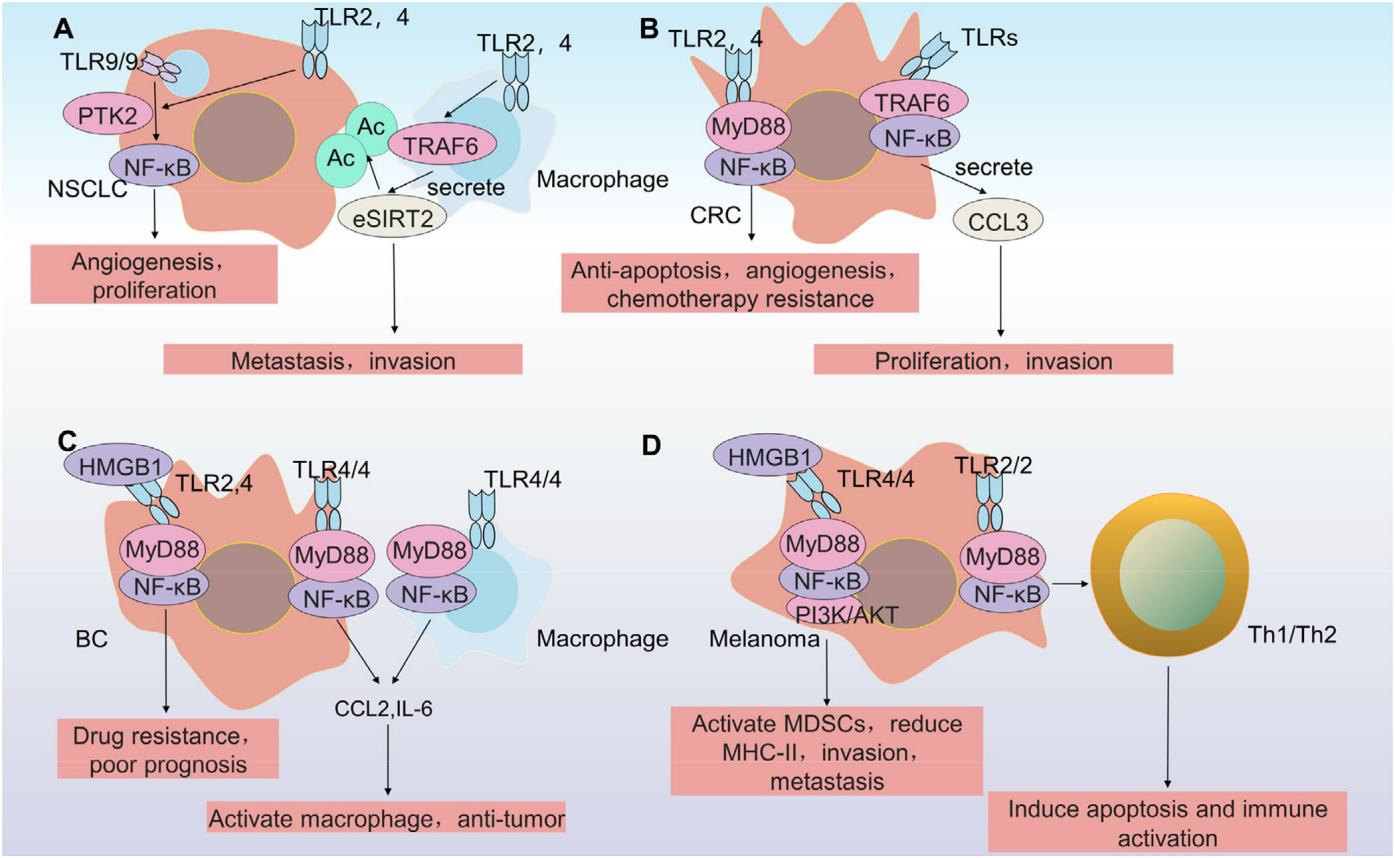
Features of TLRs in solid cancers. (A) In non‐small cell lung cancer, TLR9–PTK2 and TLR2/4 coactivation on tumor cells via NF‐κB drives angiogenesis, whereas TLR2/4–TRAF6 signaling on infiltrating macrophages results in the secretion of eSIRT2 to induce membrane protein deacetylation, concurrently promoting metastatic dissemination and stromal remodeling. (B) In colorectal carcinogenesis, dual TLR2/4‐NF‐κB activation orchestrates antiapoptotic, proangiogenic, and chemoresistance programs, triggering CCL3‐mediated autocrine/paracrine loops that amplify tumor invasiveness and niche adaptation in parallel. (C) In breast cancer, TLR2/4–NF‐κB axis activation by tumor‐derived DAMPs is correlated with chemoresistance and poor prognosis, whereas paradoxical TLR4 costimulation of tumor‐associated macrophages reprograms their polarization to establish immune‐tolerant microenvironments. (D) In melanoma progression, the dual HMGB1‒TLR4 axis activates the NF‐κB‒PI3K‒AKT cascade in myeloid‐derived suppressor cells, promoting MHC‐II suppression and metastatic spread, with concomitant TLR2 signaling in tumor cells triggering Th1‒Th2 imbalance‐mediated immunogenic apoptosis. TLR, Toll‐like receptor; NF‐κB, nuclear factor kappa B; PTK2, protein tyrosine kinase 2; TRAF6, tumor necrosis factor receptor‐associated receptor; SIRT2, sirtuin 2; CCL3, chemokine C‐C motif chemokine ligand 3; DAMPs, damage‐associated molecular patterns; PI3K, phosphatidylinositol 3‐kinase; HMGB1, high mobility group box 1; mTOR, mechanistic target of rapamycin; MHC, major histocompatibility complex; AKT, protein kinase B.

In solid tumors, TLR9 orchestrates a regulatory influence on downstream signaling pathways (Figure 3), including the NF‐κB, Src/MAPK, Wnt, and PI3K/Akt pathways, thereby controlling the initiation and progression of tumors [151, 152, 153]. Genetic ablation of TLR2, TLR4, and TLR9 in K‐ras‐driven lung adenocarcinoma leads to a reduction of tumor burden, angiogenesis, and tumor cell proliferation. Concurrently, this intervention promotes cancer cell apoptosis and remodels the tumor microenvironment to exhibit antitumor properties. Mechanistically, genetic ablation of downstream signaling components corroborates these preliminary observations through systematic quantification of TLR/NF‐κB axis activation dynamics in respiratory tract models [154].

#### 3.5.1 Lung Cancer

Protein tyrosine kinase 2 (PTK2) interacts with downstream signaling molecules of TLRs, positively regulating the TLR/NF‐κB signaling pathway. This signaling nexus confers prosurvival functions in non‐small cell lung cancer (NSCLC), as demonstrated by its indispensable role in maintaining tumor cell proliferation in vitro models [155]. Sirtuin 2 (SIRT2) serves as a crucial regulatory factor that maintains genomic stability [156]. Macrophages secrete SIRT2 after activation of TLR4 or TLR2. The translocation of SIRT2 into autophagosomes occurs in a TLR‐dependent manner. TRAF6 activates TLR2/4 via autophagic flux to release SIRT2 into the extracellular space (eSIRT2), which facilitates cancer cell metastasis and invasion. This process relies on eSIRT2‐mediated deacetylation of extracellular proteins [157]. Gene set enrichment analysis revealed that β‐arrestin 2 (ARRB2) reduced TLR3/4‐induced autophagy signaling. Concomitantly, knocking out ARRB2 significantly enhanced NSCLC invasion, progression, and metastasis [158].

#### 3.5.2 Colon Cancer

In CRC carcinogenesis, the TLR4/NF‐κB/TNF‐α axis drives proangiogenic programming through STAT3‐dependent mechanisms. Mechanistically, TLR4‐initiated signaling converges with STAT3 phosphorylation, facilitating its nuclear translocation, where STAT3 dimerizes with cofactors to transactivate tumor angiogenesis‐related genes [159]. TLR2 drives the progression of chronic inflammation in CRC tumors, and the activation of NF‐κB can prevent tumor cell apoptosis and is associated with chemotherapy resistance. Moreover, activation of the TLR pathway also elicits the secretion of chemokines and cytokines by tumors, which further promotes tumor growth and invasion [160]. For instance, the chemokine C‐C motif chemokine ligand 3 (CCL3) secreted by CRC plays an important role in the occurrence and development of tumors [35]. Protein chip analyses of CRC tissue samples, CCL3 was found to be highly expressed and closely related to the TLR/TRAF6/NF‐κB pathway, which facilitates invasion and proliferation of cancer cells [161]. CCL3 activates the TLR/TRAF6/NF‐κB axis through ligand‒receptor binding, triggering TRAF6 ubiquitination to stabilize the IKK complex, thereby driving NF‐κB nuclear translocation and transcriptional activation of proinvasive genes and cyclins [161].

#### 3.5.3 Breast Cancer

In breast cancer, TLRs are heterogeneously distributed among tumor cells, stromal cells, and infiltrating immune cells, exerting dual function: activating antitumor immunity while promoting angiogenesis to facilitate tumor escape [162]. Triple‐negative breast cancer (TNBC), a high degree of malignancy, spatial transcriptomic, and single‐cell RNA sequencing data analyses revealed that the activation of TLR4 in tumor cells enhances the drug responsiveness [163, 164]. High‐mobility group box 1 (HMGB1) functions as a DAMP and is secreted into the extracellular environment via stress differences. The binding of extracellular HMGB1 to TLR2/4 activates MyD88‐dependent NF‐κB/STAT3 signaling in breast cancer cells, driving epithelial‒mesenchymal transition and chemoresistance, while simultaneously polarizing TAMs toward the M2 phenotype. TLR2 and HMGB1 correlate with breast cancer progression and poor prognosis. Moreover, regardless of HMGB1, TLR4 is associated with a poor prognosis and chemotherapy resistance in breast cancer [165]. Although TLR4 is associated with poor prognosis in patients with breast cancer, its activation in microglia can effectively reduce the degree of brain invasion of breast cancer cells [166].

#### 3.5.4 Melanoma

In melanoma, myeloid‐derived suppressor cells (MDSCs) potently suppress the immune system and diminish the efficacy of immune checkpoint inhibitors (ICIs). HMGB1 can serve as a ligand for TLR4 in melanoma, is highly expressed in tumors, and drives MDSC activity upon activation. Single‐cell sequencing revealed that downstream inflammatory genes of TLR4/NF‐κB were enriched in the monocytes of patients, accompanied by reduced major histocompatibility complex (MHC)‐II expression—markers of immune evasion [167]. LASSO regression analysis of melanoma patients identifies TLR2 as a favorable prognostic marker in melanoma. Upon TLR2‐mediated immune response activation, melanoma cell apoptosis is induced by Th1 and Th2 activation. Conversely, activation of the TLR/PI3K–AKT pathway in melanoma promotes invasion and metastasis of melanoma cells [4]. During melanoma progression, recruitment and transformation of neutrophils into a proangiogenic state through TLR4 activation facilitate lung metastasis [168].

#### 3.5.5 Other Solid Tumors

In pancreatic cancer patients undergoing neoadjuvant therapy, TLR1, 3, and 9 are heightened expressed. Specifically, TLR1 is believed to increase the survival of patients [169]. For prostate cancer (PCa), paclitaxel resistance is the primary clinical barrier to chemotherapy, and knocking out TLR4 increases drug sensitivity and inhibits proliferation and migration, offering a mechanistic insight into resistance reversal [170]. In urothelial bladder carcinogenesis, TLR4 functions as a microenvironmental rheostat, orchestrating innate immune evasion through DAMP‐mediated autocrine signaling and paracrine reprogramming of TAMs [171]. TLR3 and TLR4 are significantly upregulated in primary esophageal cancer, gastroesophageal junction tumors, and metastatic lymph nodes [172]. Recent studies have shown that TLR5 is overexpressed in human PCa cells. Paradoxically, clinical analyses have demonstrated that elevated TLR5 expression in tumor tissues correlates with favorable survival outcomes, potentially due to its role in activating antitumor immunity through neutrophil recruitment [173].

## 4 The Application of TLRs in Tumor Treatment

A profound mechanistic understanding of TLRs in various tumors furnishes a robust a theoretical basis for TLR‐targeted tumor treatment strategies. In the following sections, we explore the application prospects and research progress of TLR agonists, antagonists, combination therapy strategies, vaccines, and other innovative modalities in oncology (Table 1).

**TABLE 1:** Characteristics of TLR‐mediated immune responses in different cell types across various tumor types.

TLRs	Cancer type	Cell type	Mechanism
TLR1–TLR2	Thyroid cancer	Tumor cell	The activation of heterodimers as tumor driver genes can stimulate the secretion of immunosuppressive cytokines such as IL‐10, helping tumors escape immune surveillance [174].
	NSCLC	Macrophage	It can drive macrophages to polarize toward M1 type through the TLR1/2‐JNK signaling pathway, exerting the function of killing tumors [175].
	CRC	DC cell	Promote the infiltration of cytotoxic lymphocytes, mediate the presentation function of DC cells, and reduce tumor burden [176].
TLR2	GC	Tumor cell	DAMP in TME activates the TLR2‐STAT3 signaling pathway in gastric cancer, promoting malignant growth and aiding distant metastasis of the tumor [177].
	NSCLC	Tumor cell	By activating TLR2 signaling and secreting inhibitory molecules such as arginase 1 and eSIRT2, it promotes malignant growth of lung cancer [157, 178, 179].
	BC	Tumor cell and macrophage	HMGB1 acts as an endogenous DAMP to activate tumor TLR2 and promote malignant development of tumors. But in macrophages, TLR2 can promote polarization toward M1 type and enhance antitumor immune response [180, 181].
TLR6–TLR2	NSCLC	Tumor cell	The expression level significantly increased in lung adenocarcinoma cell lines, which may be related to the tumor promoting effect of tumor associated neutrophils [182].
	CRC	Colonic epithelial cells	High expression of TLR2–TRR6 heterodimer in colonic epithelial cells, when activated by DAMP, can increase the expression of tumor suppressor factors such as glycoproteins and T‐cell antigen‐1 (TIA1), thereby preventing tumor progression and invasion [183].
TLR3	BC	Tumor cell	TLR3 plays a dual role in breast cancer. On the one hand, TLR3 inhibits the malignant development of tumor cells by interacting with cancerous signaling pathways; on the other hand, TLR3 can promote breast cancer cells to differentiate into tumor stem cells, which is not conducive to tumor prognosis [184, 185].
	TNBC	DC cell and macrophage	TLR3 can mediate the maturation and differentiation of DC cells. In TNBC, DC cells recognize tumor components through TLR3 and exert immune surveillance function through DAMP [186]. The activation of TLR3 signaling on macrophages in TNBC tissue can mediate an increase in the secretion of chemokines such as CXCL10, inflammatory cytokines IL‐6, TNF‐α, and promote antitumor immune response [187].
	NSCLC	Tumor cell	The activation of TLR3 helps tumor cells evade immune surveillance and promotes tumor metastasis [151, 158].
	AML	Macrophage	The activation of TLR3 pathway in macrophages can activate macrophages and counteract the antiphagocytic effect of AML cells through CD47 [188].
TLR4	NSCLC	Tumor cell	TLR4 is activated through expression IL‐6, chemokine CCL2, VEGFA, inducing tumors escape and metastases [189].
	TNBC	Macrophage	The activation of TLR4 in macrophages can promote the enhancement of macrophage presentation function and the chemotaxis of cytotoxic lymphocytes to exert the function of killing tumor cells [163].
	Lymphoma	B cell	Lymphoma cells secrete TNF‐α, which enhances TLR4‐mediated signaling in intestinal B cells and in turn inhibits malignant growth of lymphoma [190].
	Cutaneous T‐cell lymphoma (CTCL)	Macrophage	Elevated expression of TLR4 protein in macrophages in CTCL. Activation of TLR4 assists in malignant invasion of tumor cells [191].
TLR5	CRC	CD8+T cell	The symbiotic microbiota in CRC patients activates CD8+T cells TLR5, promoting T cell cytotoxicity against tumors [192].
	TNBC	Tumor cell	Components of tumor cell activate TLR5 signaling, leading to increased secretion of IL‐8 cytokines and promoting malignant development of tumors [193].
TLR7	BC	Tumor cell, macrophage, DC cell	Dying tumor cells release ssRNA to activate TLR7 signaling, which leads to increased tumor invasiveness [194]. However, the activation of TLR7 of infiltrating macrophages in breast cancer will reduce the secretion of inhibitory inflammatory factor IL‐10, thus promoting the infiltration of cytotoxic lymphocytes into tumor tissue [195]. Activation of TLR7 signaling in DC cells increases the expression levels of various factors that promote antitumor immune response, including IFN‐γ and TNF‐α [196].
	NSCLC	Tumor cell	The activation of TLR7–IFN signaling in lung cancer cells leads to an increase in the secretion of chemokine CXCL10, which contributes to immune cell infiltration [197].
	CRC	DC cell	The activation of TLR7 signaling in DC cells can effectively promote Th1 type immune response and inhibit Treg cells [198].
	AML	DC	TLR7 can activate Th1 cells in DC cells by mediating the IFN–STAT1 signaling pathway, mediating the immune response against AML and preventing tumor cells from escaping immune surveillance [138].
TLR8	AML	Tumor cell	In AML, activation of TLR8 can lead to caspase‐3‐dependent apoptosis of tumor cells, which mediates antitumor effects [199].
	NSCLC	Tumor cell	The expression level of TLR8 increases in lung cancer cells, and TLR8 is one of the factors contributing to poor prognosis of lung cancer [200, 201].
TLR9	CRC	Tumor cell	The activation of TLR9 in tumors can mediate the upregulation of IFN‐γ expression, promote the activation of immune cells such as B and T cells, and exert the effect of killing tumors [202, 203].
	HCC	Tumor cell	Hypoxia or histone modification can activate TLR9 signaling in tumors, thereby inducing TLR9 caspase‐1 activation and promoting malignant development of tumors [204].
	NSCLC	Cancer stem‐like cells (CSC)	When mitochondrial autophagy occurs in tumor cells, channel proteins open and release mitochondrial DNA into the cytoplasm, which is recognized by TLR9. The activation of TLR9 interacts with the signaling pathway of CSC cell expansion, promoting tumor drug resistance and recurrence [205].
	AML	DC cell	The activation of TLR9 signaling in DC cells can upregulate the expression levels of cytokines such as TNF‐α and IL‐6 that mediate antitumor immune responses [206].
	ABC‐DLBCL	Tumor cell	In ABC‐DLBCL, TLR9 and B cell receptors together constitute tumor driver genes. TLR9 activates various cytokines including IL‐10, and has cross‐linking effects with multiple malignant signaling pathways [150].

Abbreviations: ABC‐DLBCL, activated B‐cell‐like diffuse large B‐cell lymphoma; AML, acute myeloid leukemia; BC, breast cancer; CCL2, (C‐C motif) ligand 2; CRC, colon cancer; CXCL10, C‐X‐C motif chemokine ligand 10; DAMP, damage‐associated molecular pattern; DC, dendritic cells; GC, gastric cancer; HCC, hepatocellular carcinoma.; HMGB1, high mobility group box 1; IFN‐γ, interferon‐γ; IL, interleukin; JNK, c‐Jun N‐terminal kinase; NSCLC, non‐small cell lung cancer; SIRT2, sirtuin 2; STAT, signal transducer and activator of transcription; TLRs, Toll‐like receptors; TME, tumor microenvironment; TNBC, triple‐negative breast cancer; TNF‐α, tumor necrosis factor‐alpha; VEGFA, vascular endothelial growth factor A.

### 4.1 TLR Agonists

The combination of TLR agonist therapies has emerged as a cornerstone in cancer immunotherapy [207]. TLR agonists mainly orchestrate immune activation by binding to their respective receptors, thereby promoting downstream secretion of inflammatory cytokines [208]. Among these, TLR7 and TLR8 agonists exhibit the broadest clinical translation in oncology [138]. In a seminal study, whole blood of patients with pancreatic cancer (PDAC) and those with intraductal myxoid tumor (IPMN), a precancerous lesion of PDAC, were plated and cocultured with R848 (a TLR7/8 agonist) for 6 h. R848 treatment induced a negative correlation between TNF secretion by peripheral blood monocytes and tumor progression grade [209]. Delivery of TLR7/8 and TLR9 agonists to primary and metastatic tumor sites has been shown to significantly reduce tumor burden and prolong the survival of patients with breast cancer [210, 211]. Additionally, novel TLR7/8‐targeted agonists demonstrate potent tumor burden reduction in xenograft mouse models [212].

TLR agonists potently activate immune cell cytotoxicity. When NK cells isolated from patients with acute lymphocytic leukemia are treated with multiple TLR agonists, namely, Poly I:C (a TLR3 agonist) and imiquimod (IMQ) (a TLR2 agonist), these agents effectively trigger the activation of NK cells. This activation is manifested by an increase in the expression of NK cell activation markers, such as CD107a and IFN‐γ. Additionally, treatment with R848 and ODN2006 (TLR9 agonists) further augments the cytotoxicity of NK cells [213].

TLR7/8 agonists primarily leverage downstream cytokines and chemokines, including IFN‐γ and TNF‐α, to enhance the function of CD8+ T and NK cells to recognize tumor cells and exert cytotoxic effects [214, 215]. In PCa cells, the TLR1/2 and TLR3 agonists L‐pampo induce reactive oxygen species (ROS) through the PI3K and STAT pathways. This oxidative stress culminate in tumor cell demise [216].

The novel TLR agonist rMBP NAP demonstrates 77.72% tumor inhibition rate in B16 melanoma mouse models [217]. Through integrated NanoString transcriptome and proteomic flow cytometry analysis, the TLR3 agonist rintatolomod has been shown to effectively activate type 1 conventional dendritic cells (cDC1s) and T cells in PDAC patients, translating to improved clinical outcomes [218].

### 4.2 TLR Antagonists

The roles of TLRs in the complex process of tumor development are diverse. Not all TLRs suppress tumor growth. While certain TLRs with tumor‐promoting properties can be targeted to enhance the efficacy of immunotherapy and reverse chemotherapy resistance. For instance, TLR2 inhibition in cancer stem cells (CSCs) has emerged as a viable strategy for achieving therapeutic endpoints while exerting negligible immunological off‐target effects [180]. PDAC cells sustain tumor inflammation and chemotherapy resistance through upregulating TLR2 and TLR9 expression. Targeted antagonism of these receptors has been shown to potently inhibit PDAC cell growth [219]. TLR4 is a predictive marker for tumor proliferation and invasion, with spasstolonin B (SsnB) inhibition of TLR4 promote the development and invasion of human PCa cell lines LNCaP, DU‐145, and PC‐3 [220, 221]. Inosine monophosphate dehydrogenase inhibitors exert immunosuppressive effects by inducing hyperactivation of TLR4/TRAF6/NFκB signaling and upregulation of the adhesion molecule VCAM1. These inhibitors have been shown to be effective in the treatment of invasive leukemia and AML with MLL rearrangement [222, 223].

### 4.3 Combination Therapy Strategies

Although tumor immunotherapy has shown promising clinical efficacy, clinical limitations persist, including suboptimal host response rates, insensitivity to immunotherapy, and systemic side effects, that compromise overall therapeutic outcomes. Owing to the remarkable immune activation properties of TLRs, their combination with immunotherapy has shown promising potential for improving objective response rates [224, 225, 226, 227]. Some TLR agonists combined with immunotherapy drugs have demonstrated dual mechanisms: inducing tumor cell apoptosis and promoting lymphocyte infiltration for direct tumor cytolysis. For instance, the poly ADP ribose polymerase inhibitor talpazopanib acts as a TLR3/9 agonist coadjuvant, elevating caspase‐3 and caspase‐8 levels in TNBC cells to induce apoptosis [228]. Human serum albumin‐encapsulated black phosphorus quantum dots (BPQDs@HSA) significantly activate the TLR‐5,9/NF‐κB pathway in NK cells by modulating the immune microenvironment. This activation facilitates NK cells exert their antitumor effects [229]. The combination of the STING agonist CDN and the TLR4 agonists MPL‐A or R848 enhance immune cell infiltration and cytotoxicity in B16 melanoma and MOC1 head and neck cancer models [230]. The combination of a TLR3 agonist and ICI, T‐cell‐activated V‐domain immunoglobulin inhibitor, induces macrophage polarization in vitro, prolonging survival in mouse bladder cancer models [231]. The coupling of the PI3K/mTOR dual inhibitors BEZ235 and R848 significantly enhance the cytotoxicity of immune cells in a mouse AML model and markedly mitigate immunosuppressive effects, while boosting the secretion of antitumor and inflammatory cytokines by immune cells [232]. Herbal‐derived melanin functions as an adjuvant for the TLR4 pathway, promoting secretion of the gastric protective markers PGE2 and IL‐6 in gastric epithelial cells [233].

### 4.4 Combination Therapy With ICIs

As mentioned in the previous section on signaling pathways, the interactive regulation between TLR signaling and inhibitory immune markers has spurred intensive research into TLR‐targeted strategies for sensitizing ICI therapies [234]. In PTEN‐deficient NSCLC exhibits a reduced response to ICI therapy, whereas TLR3 and TLR7/8 agonists Poly (I: C) and R848 enhance anti‐PD1 therapy by suppressing regulatory T (Treg) cells [207]. Using nanoprogrammable switches, c‐N@IM/JQ encapsulates IMQ (TLR7/8 agonist), which can be automatically released into the tumor area. By activating TLR signaling, this system downregulates PD‐L1 expression, thereby enhancing cytotoxic T cell‐mediated tumor lysis [235]. A tumor vaccine composed of a TLR7/8 agonist sensitize the therapeutic effect of anti PD1 in bladder cancer and melanoma mouse models [236]. In a mouse T‐cell lymphoma cell model, coadministration of TLR3 and TLR9 agonists combined with anti‐CTLA‐4 and anti‐PD1 elicit robust antitumor effects, augmenting the efficacy of tumor vaccines [237]. The combined therapeutic effect of TLR agonists and ICI has been validated in a mouse model of breast metastatic cancer [210]. When conducting proteomic and transcriptomic multiomics analysis of the serum of PDAC patients treated with the TLR3 agonist rintatolimod, it was found that activation of TLR3 effectively reduces the expression of PD‐L1 and lower immune tolerance [218]. In melanoma models, the combination of TLR agonists and ICI therapy in melanoma models requires B cells involvement, with B cells enhancing the therapeutic efficacy of anti‐PD1/R848 cotreatment [238]. A TLR7 agonist combined with anti‐PD‐L1 therapy in a mouse model of colorectal cancer effectively reduce the tumor burden and inhibit distant metastasis of tumors [224].

### 4.5 Application of CRISPR/Cas9 Technology in TLRs

The discovery of CRISPR/Cas9 technology has revolutionized biological research, as it enables precise disruption of specific sequences through the guidance of sgRNA and has also been pivotal in the field of TLR research [239]. CRISPR/Cas9‐mediated gene editing in mouse B cell research showed that TLR–IRF7 signaling is regulated by the TF IRF8, expanding the mechanistic understanding of TLR signaling cascades [240]. Desmosome knockout cell lines exhibit heightened sensitivity to TLRs–NF‐κB signaling, and this response has shown to be remedied by NF‐κB inhibitors, establishing desmosomes as novel regulators of TLR signaling [241]. TLRs are considered to promote malignant tumor invasion along with one of the driving genes in CLL. However, knocking out IRAK4 and MyD88 using CRISPR/Cas9 technology did not have a remedial effect on tumor proliferation, demonstrating that TLR signaling is not a primary oncogenic driver [144]. Knockout of PTK2 in lung cancer models reveals a crosstalk between TLRs and other signaling molecules in lung cancer and provides a rationale for TLR‐driven targeted therapy of malignant tumors [155]. A nanodelivery system to deliver PD‐L1‐targeted CRISPR/Cas9 and TLR2 agonists into HCC tissues effectively increases cytotoxic lymphocyte infiltration, outperforming single‐agent ICI therapy in preclinical models [226].

### 4.6 Nanomaterial‐Based Synergistic Strategies for TLR‐Based Therapy

**FIGURE 4:**
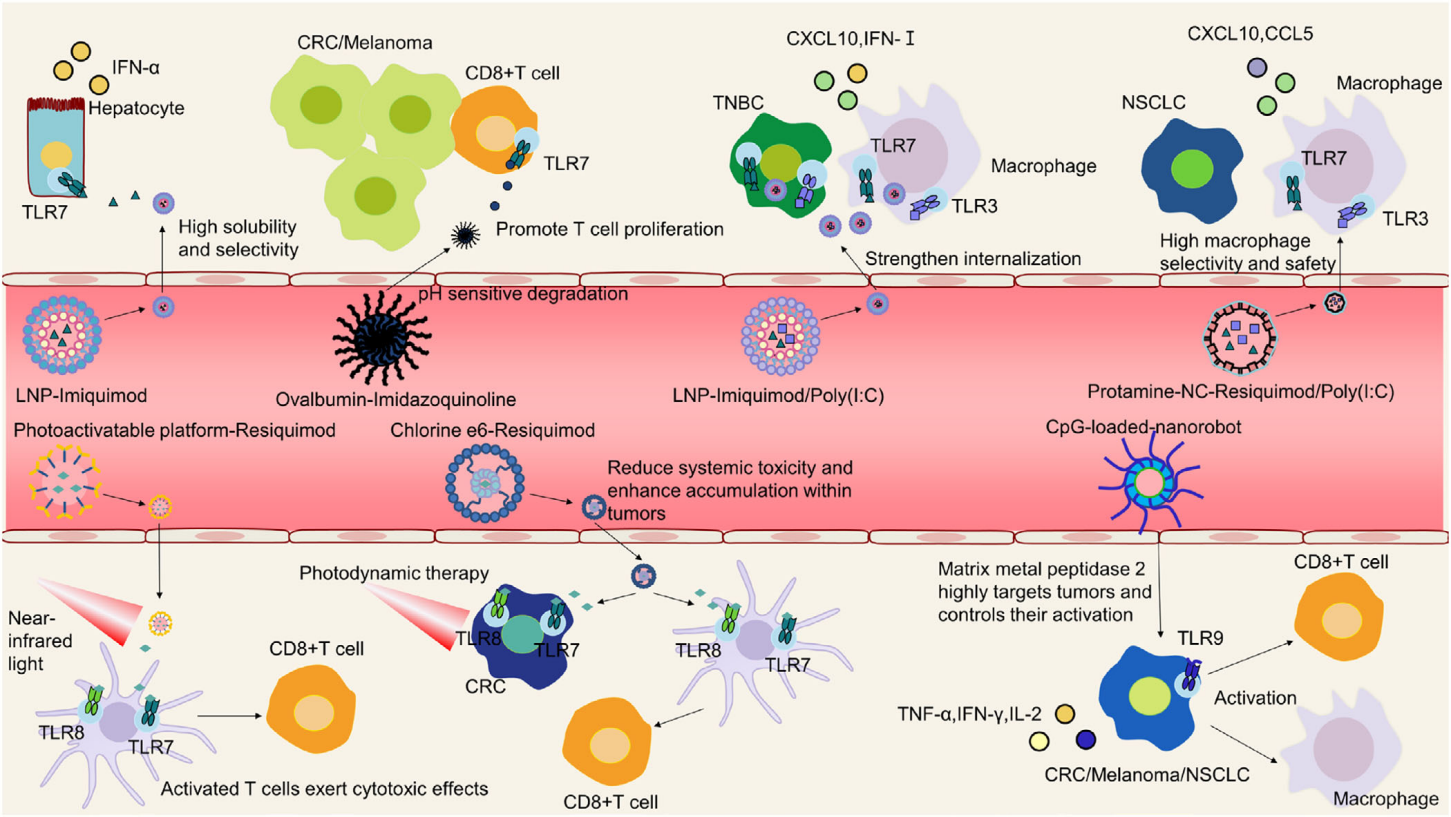
Schematics of nanocarrier‐based TLR agonist delivery systems for cancer immunotherapy. LNP‐encapsulated imiquimod: This system utilizes LNPs to encapsulate the TLR7 agonist imiquimod. Following intravenous administration in a hepatocellular carcinoma mouse model, it selectively targets hepatocytes. Activation of endosomal TLR7 receptors on hepatocytes triggers the TLR–IFN signaling pathway, upregulating IFN‐α expression and initiating antitumor immune responses. Ovalbumin‐conjugated imidazoquinoline: Ovalbumin is covalently linked with TLR7 agonist imidazoquinoline. Tail‐vein injection in colon cancer and melanoma mouse models enables pH‐responsive degradation, specifically targeting endosomal TLR7 receptors on CD8 T cells. This activates antitumor functions of CD8 T cells, promotes their proliferation, and significantly reduces tumor burden. LNP‐encapsulated Imiquimod and poly(I:C) dual agonists: LNPs deliver a combination of TLR7 agonist imiquimod and TLR3 agonist poly(I:C) for triple‐negative breast cancer treatment. Intravenous administration ensures high safety and specificity, enhancing cellular internalization. Activation of TLR7 and TLR3 in macrophages and tumor cells upregulates CXCL10 and IFN‐I, potently activating antitumor immunity. Protamine‐nanoparticle‐encapsulated resiquimod and poly(I:C) dual agonists: Protamine nanoparticles encapsulate TLR7 agonist resiquimod and TLR3 agonist poly(I:C) for non‐small cell lung cancer therapy. With high macrophage selectivity and safety, this system increases agonist accumulation in macrophages. TLR3/TLR7 activation upregulates CXCL10 and CCL5, driving antitumor immune responses. Photoactivatable platform‐resiquimod: A light‐responsive platform delivers resiquimod TLR7 to tumor tissues. Following specific targeting of DCs, near‐infrared irradiation triggers agonist release, then activating endosomal TLR3/TLR7 on DCs. This process enhances antigen presentation, promotes DC migration to lymph nodes, and stimulates CD8⁺ T cell proliferation and cytotoxicity against tumors. Chlorine e6–resiquimod cholesterol conjugate resiquimod: Cholesterol‐based formulation improves cell affinity, solubility, and safety of resiquimod. Targeting colon cancer cells and DCs, this system reduces off‐target toxicity. Combined with photodynamic therapy, it induces tumor cell death and releases DAMPs, further activating TLR‐mediated immune responses in DCs to prime CD8⁺ T cell‐mediated tumor killing. MMP2‐responsive nanorobots loaded with CpG DNA: Nanorobots functionalized with MMP2 carry the TLR9 agonist CpG DNA, enabling tumor‐specific targeting and controlled agonist release. In multiple tumor models, TLR9 activation in tumor cells increases secretion of TNF‐α, IFN‐γ, and IL‐2, activating immune cells and remodeling the tumor microenvironment to inhibit recurrence. Each panel illustrates the nanomaterial composition, targeting mechanism, TLR receptor activation, signaling pathways, and therapeutic outcomes in preclinical cancer models, highlighting diverse strategies for enhancing TLR agonist delivery and antitumor immunity. LNP, lipid nanoparticle; TLR, Toll‐like receptor; NF‐κB, nuclear factor kappa B; IFN, interferon; CXCL10, C‐X‐C motif chemokine ligand 10; CCL5, chemokine C‐C motif chemokine ligand 5; DCs, dendritic cells; DAMPs, damage‐associated molecular patterns; MMP2, matrix metallopeptidase 2; TNF‐α, tumor necrosis factor‐alpha; IL, interleukin.

TLR‐targeted therapy has promising therapeutic efficacy; however, clinical translation is hindered by dose‐limiting toxicities, poor pharmacokinetics, and insufficient tissue tropism (Figure 4). In this context, nanomaterial‐based transport systems have emerged as transformative platforms for TLR‐related therapies [242, 243]. For instance, a polybutamine (PDA) calcium carbonate‐based delivery system, which involves the TLR7 agonist IMQ and CD3 immune antibody OKT3, enabling effectively deliver drugs to the cancer site in a mouse model of breast cancer. This system suppresses the activity of Treg cells while augmenting immune effector cell cytotoxicity [244, 245]. Liposome nanoparticle‐encapsulated TLR7 agonist IMQ and TLR3 agonist (poly I:C, IC) were delivered to tumor tissue, promoting macrophage polarization and the secretion of type I IFN and CXCL10, thereby converting “cold” tumors into “hot” tumors and improving ICI treatment efficacy [246]. Proton polymer nanocapsules containing poly (I:C) and R848 promote TAM polarization and effectively reduce the tumor burden after intravenous injection in tumor‐bearing mice with negligible systemic toxicity [235, 247]. Researchers have designed a photoactivatable nanosystem (PNA) that generates near‐infrared (NIR) light‐induced immunogenic cell death (ICI) in dying tumor cells while simultaneously releasing R848. In a mouse model, PNA generated ROS to kill tumors and induced ICI under infrared light irradiation, gradually releasing R848. In preclinical studies, the injection of PNA effectively reduced the tumor burden and significantly activated the antitumor T‐cell response [248]. A nanograded polymer composed of a Ce6 photosensitizer and R848 induces ICI through photodynamic therapy. This polymer has low systemic toxicity when intravenously injected into a subcutaneous colorectal cancer model and effectively inhibits tumor growth [249]. R848 is carried by pH‐sensitive nanoparticles wrapped in paclitaxel and L'Aquilomo, which cause breast cancer cells to die and induce macrophage polarization [250]. A TLR9/TLR7/8 dual‐agonist delivery system, CPG@Au NRs/m‐R848, in combination with photothermal immunotherapy, effectively inhibits tumor development in melanoma models by promoting TAM polarization and DC maturation [251]. Although TLR7/8 agonists have shown to effectively enhance immune therapy, as initiating receptors of innate immunity, they may cause severe cytokine storms and endanger the lives of patients. In order to overcome this problem, researchers used degradable gel, and gel was slowly explained in vivo, so that TLR7/8 agonist was continuously released in a small amount in vivo, effectively increasing its safety while maintaining antitumor efficacy in melanoma and breast cancer models [252].

Through electrostatic interactions, polyanionic alginate and polycationic polyethyleneimine deliver TLR9 agonist unmethylated CpG adjuvant to tumor models, stimulating DC maturation and induces cytotoxic T cells activation with low systemic toxicity [253]. The extension of the half‐life of IL‐2 super factor H9‐MSA (mouse serum albumin) combined with the TLR9 agonist CpG ODN resulted in delayed growth and prolonged survival in a B16‐B2m−/− mouse model [254]. In a TLR9‐positive B16 tumor model, intelligent tumor microenvironment‐responsive nanorobots were used to deliver CpG to induce autophagy in the tumor cells. This delivery system significantly induced massive cell death in the B16 cells (IC50 = 12.0 µg/mL). However, this system has poor therapeutic effects on TLR9‐negative cells [255].

Trace amounts of Mn^2+^ ions bind to bovine serum albumin (BSA), thereby forming Mn@BSA nanocomposites, activating TLR4 signaling in melanoma models, and initiating antitumor immune responses. The underlying mechanism of this system involves the activation and polarization of TAMs, which in turn inhibit tumor growth [256].

### 4.7 TLR Vaccines

Cancer vaccines represent immunologically engineered therapeutics that harness the capacity of the immune system to target tumors. Utilizing antigens to activate immune cells enables the elimination of tumor threats. Moreover, a subset of activated cells establishes long‐term persistence over an extended period and expand upon exposure to antigens [257]. The PAMP/DAMP signal triggers innate immune cells to initiate persistent adaptive immunity, a critical prerequisite for cancer vaccines [258, 259, 260]. The first candidate human vaccine, ATP128, which was created based on the KISIMA platform, promotes DC activation through TLR agonist‐derived peptides. This vaccine carries CRC cell tumor‐associated antigens, which can be delivered through cell‐penetrating peptides and activates the TLR2, 4/NF‐κB, and IRF3 pathways, thereby upregulating inflammatory cytokines, IFNs, and MHC class I transcription [261].

Using computer‐aided molecular design and machine learning, researchers constructed a library of 46 gold nanoparticle (AuNP) adjuvants functionalized with TLR agonist ligands. Among these, AuNPs27 and 35 promoted DC activation and antigen presentation. These two vaccine adjuvants enable activation of multiple TLRs, significantly enhancing DC activation and T cell expansion, accompanied by a marked increase in downstream expression of IL‐12 [262]. A vaccine formulated with TLR7/8 agonist epitopes effectively and accurately introduce antigens and vaccine adjuvants via self‐assembly and carrier‐free nanovaccines. Compared with conventional vaccines, activation of the TLR signaling pathway promotes DC activation and results in increased local retention time and lymph node drainage efficiency, eliciting robust CD8+ T‐cell responses and demonstrating remarkable therapeutic efficacy in melanoma models [263]. Transport of cancer vaccine‐associated antigen mucin 1 (MUC1) and TLR7/8 agonists via C3 liposomes alleviates MUC1 resistance. This treatment approach significantly activates the antigen‐specific immune response, leading to increase the T‐cells activation and expansion [264]. Using virtual simulation and immune informatics tools, researchers have constructed a 3D‐structured vaccine vector to model TLR7/8, with molecular docking, molecular dynamics simulation, and MMBPSA confirmed the stability of the TLR complex in the vaccine, and its immunogenicity‐driven immune responses [265].

mRNA vaccines have emerged as promising candidates for their excellent delivery ability and their ability to enhance immune responses in pancreatic ductal adenocarcinoma. Docking analysis of mRNA vaccines revealed that they can bind to TLR2/4 and increase the differentiation of memory B and T cells, as well as increase the levels of helper T cells and immunoglobulins (IgM and IgG) [266]. Adding the TLR2/6 agonist Pam2Cys adjuvant to mRNA lipid nanoparticles (LNPs) induce surges in the levels of IL‐1β, TNF‐α, and MIP‐1β in C57BL/6 mouse serum 24 h after injection. Enhanced antigen cross‐presentation promotes cytotoxic CD8+ T‐cell infiltration, with flow cytometry revealing granzyme B+ tumor‐infiltrating lymphocytes in E.G7–OVA models. This combinatorial effect resulted in 50% established tumor eradication in the OVA peptide‐vaccinated cohorts [267].

### 4.8 Clinical Trial

Clinical trials represent the gold standard for evaluating a treatment method. In the previous section, we provided a detailed introduction to research on TLR agonists and antagonists in the field of cancer treatment. Next, we elaborate on the clinical trials of TLR agonists in the field of cancer treatment in recent years (Table 2). In a randomized phase II clinical study, the safety and efficacy of the combination therapy of resiquimod (TLR7/8 agonist) and Poly (I: C) (TLR3 agonist) with a tumor vaccine targeting DC cells were evaluated in patients with malignant glioma. Through single‐cell analysis, it is known that this combination activates the TLRs–IFN signaling pathway in immune cells of patients, thereby exerting a tumor killing effect and ensuring safety [275]. In another phase I/II clinical study, Poly (I: C) and resiquimod combined with tumor vaccines were also used to evaluate their efficacy in patients with advanced melanoma. The results indicated that the combination of Poly (I: C) enhanced lymphocyte sensitization [273]. IMQ (TLR7 agonist) and radiology was tested in a phase I/II clinical trial for metastatic breast cancer. Posttreatment sample analysis showed, compared with the control group, the transcription of CCL5, GZMB, and NK cell activation pathways was upregulated, and the metastasis of breast cancer was effectively controlled, which proved the feasibility of TLR7 agonist combination therapy in the treatment of clinical metastatic breast cancer [276]. IMQ has been used as an immune adjuvant in patients with advanced melanoma and has been shown to enhance the immune response and strengthen lymphocyte infiltration, suggesting its potential in further clinical treatment research [277]. In an early phase I clinical trial of patients with OSCC, IMQ showed a significant increase in cytotoxic lymphocytes and a significant reduction of tumor cells after pathological examination, correlating with a favorable good prognosis, indicating its therapeutic effect in OSCC [278]. G305 (TLR4 agonist) and anti‐PD‐L1 therapy were evaluated for their efficacy in a phase II clinical trial of synovial sarcoma or mucinous liposarcoma. Although no significant changes in PFS were observed, pathological examination showed a significant increase in CD8+ T cell infiltration, suggesting an immune sensitization effect [279]. In ac I clinical trial, Poly (I: C) was used in combination with tumor vaccines and ICI therapy in patients with advanced melanoma. The trial results showed a significant increase in enhanced intratumoral T‐cell proliferation and activation [280]. In a phase I clinical trial of advanced esophageal cancer patients, the combination therapy between Poly (I: C) and tumor vaccine was also used, and the result showed a significant increase in antibody titers [281]. The same treatment method yielded similar results in patients with melanoma. In another phase I/II clinical trial, the Poly (I: C) combination therapy group effectively activated the immune response of CD8+ T cells to tumor cells, and the safety was also guaranteed [282]. Another TLR7 agonist, LHC165, was used in combination with anti‐PD‐1 therapy in a phase I clinical trial. After receiving intratumoral injection in neck squamous cell carcinoma patients presented higher safety and antitumor efficacy [283]. The TLR3 agonist rintatolomod serve as an immune adjuvant for ICI therapy. A clinical trial in phase I/II PDAC patients showed that the combination of rintatolomod and durvalumab (anti‐PD‐L1) can activate DC cells and T cells, with potential antitumor effects.

**TABLE 2:** Clinical trials of TLR agonists in cancer therapy.

TLR agonist or antagonist	Combined therapy	Conditions	Phase	NCT	Status	Potential	Result or failure reason	Reference
Rintatolimod (TLR3 agonist)	Durvalumab (anti‐PD‐L1)	Metastatic pancreatic ductal adenocarcinoma	I/II	NCT05927142	Recruiting	Activate immune cells using rintatolomod to evade resistance to ICI therapy in metastatic pancreatic ductal adenocarcinoma	NA	[268]
CAN1012 (TLR7 agonist)		Metastatic advanced solid tumors	I	NCT04987112	Recruiting	Evaluate the effectiveness of intratumoral injection	NA	
TransCon™ resiquimod (TLR7/8 agonist with a carrier)	TransCon™ IL‐2 β/​γ	Advanced or metastatic solid tumor	I/II	NCT05081609	Recruiting	Using TransCon carrier to slowly release drugs and improve their safety	NA	[269]
Resiquimod (TLR7/8 agonist)	gp100 and MAGE‐3 (tumor vaccine)	Melanoma	II	NCT00960752	Completed	Activate cytotoxic lymphocytes to kill tumor cells by activating TLRs and assist lymphocytes in recognizing tumor cells through tumor vaccines	Resiquimod activates various immune cells, including DC cells and cytotoxic lymphocytes, to reduce the burden of metastatic melanoma tumors.	[270]
Poly(I:C) (TLR3 agonist)	CDX‐1140 (anti‐CD40) and 6MHP and NeoAg–mBRAF (tumor vaccine)	Melanoma	I/II	NCT04364230	Completed	The combination of vaccines and immune agonists exerts specific immune activation function.	No result	
MEDI9197 (TLR7/8 agonist)	Durvalumab (anti‐PD‐L1)	Solid tumors	I	NCT02556463	Terminated	MED19197 can effectively activate antitumor immune response in mouse models and has great potential in combination with ICI therapy.	Although it can activate the patient's immune response, the incidence of adverse reactions increases.	[271]
EIK1001 (TLR7/8agonist)	Pembrolizumab (anti‐PD‐1) and chemotherapy	Advanced melanoma	II/III	NCT06697301	Recruiting	EIK1001 has the ability to activate antitumor immunity, but its safety and tolerability are still unknown.	NA	[272]
EIK1001 (TLR7/8agonist)	Pembrolizumab(anti‐PD‐1) and chemotherapy	NSCLC	II	NCT06246110	Recruiting		NA	
Poly (I:C) and resiquimod (TLR3 and TLR7/8 agonist)	Peptide vaccine (LPV7) + etanus peptide (tumor vaccine)	Melanoma	I/II	NCT02126579	Completed	Activation of T cell‐mediated antitumor immune response using TLR agonists	The safety of combination therapy has been confirmed, and Poly (I: C) can significantly activate immune cells.	[273]
BDB018 (TLR7/8 agonist)	Pembrolizumab (anti‐PD‐1)	Solid tumor	I	NCT04840394	Completed	BDB018 is a new generation TLR agonist with better efficacy and safety.	No result	
DV281 (TLR9 agonist)	Nivolumab (anti‐PD‐1)	Advanced NSCLC	I	NCT03326752	Completed	Preclinical studies have confirmed that the novel TLR9 agonist has good safety and antitumor immune activation function and has potential therapeutic value in NSCLC patients.	No result	
VTX‐2337 (TLR8 agonist)	Nivolumab (anti‐PD‐1)	Heck and neck cancer	I	NCT03906526	Terminated	Preclinical studies have confirmed that VTX‐2337 has a potent effect on activating immune cells and can also enhance sensitivity to ICI therapy.	After treatment, the maturation of immune cells increased and lymphocyte infiltration increased. However, due to the small size of the queue, statistical differences could not be obtained, and single‐cell analysis did not reveal activation of TLR8 signaling.	[274]
Emavusertib (TLR downstream signaling antagonists)	Venetoclax (Bcl‐2 antagonist)	Acute myelogenous leukemia	I/II	NCT04278768	Recruiting	TLRs are commonly used as malignant driving proteins in AML, and blocking their downstream signaling molecules can effectively reduce the secretion of proinflammatory cytokines, which has the potential to treat AML.	NA	

Abbreviations: AML: acute myeloid leukemia.; ICI: immunogenic cell death; NSCLC: non‐small cell lung cancer; TLRs: Toll‐like receptors.

The above synthesizes clinical trials of TLRs agonists in the past 5 years. Overall, the combination of TLRs agonists and immunotherapy have exhibited promising therapeutic effects, underscoring the potential of TLRs agonists as adjuvants in immunotherapy. However, most clinical studies still have limitations. Most of the researches focus on the study of immunotherapy markers, rather than patient survival rates. Besides, many studies have too few patients entering the queue so that lack of persuasiveness. Moreover, TLRs inhibitors have not received enough attention, and the selection of TLRs agonists is too limited. Future research should be conducted to explore the clinical effects of different TLRs. Overall, TLRs agonists and inhibitors still have promising prospects in clinical research.

## 5 Conclusion and Prospects

Tumor immunotherapy has advanced substantial progress in recent decades, owing to research breakthroughs on TLRs. This activity profoundly shaped our understanding of tumor pathogenesis and therapeutic strategies. The applications of TLR agonists, antagonists, delivery systems, and vaccines have facilitated the exploration of numerous avenues for cancer treatment. Concurrently, with the growing interest in tumor microbiota, the ability of TLRs to detect microbial PAMPs within tumors has garnered increasing attention [284, 285, 286, 287].

Distinct from most other reviews of TLRs, we have elucidated the different functions of TLRs in different cell subpopulations at the mechanistic level, focusing on signaling mechanisms such as chromatin remodeling, chromatin acetylation, TLR subgroup specificity, and interactions between TLR signaling pathways and other signaling networks. These studies on signaling mechanisms are at the forefront of research on TLRs. In subsequent chapters, we summarized the latest research on TLRs in the context of tumors, including the impact of the gut microbiota balance and SNPs in popular fields. We classify common malignant tumors into three subgroups: microbiota‐related, lymphatic hematopoietic system tumors, and solid tumors. This taxonomy is based on the special function of TLRs, because TLRs, as members of the PRR family, can not only recognize tumor components, but also capture the specific structure of pathogenic microorganisms. Therefore, in the context of many tumors caused by pathogenic microorganisms (such as GC, cervical cancer, and liver cancer), TLRs exhibit immune responses different from those of solid tumors. Invasive pathogenic microorganisms even selectively inhibit TLRs to achieve immune evasion. In lymphatic hematopoietic system tumors, malignant immune cells themselves are rich in TLRs, which should serve as a pathway for immune cells to monitor tumors but play a role in driving malignant progression. Therefore, there are many differences between this subgroup and solid tumors. Within the same subgroup, identical TLRs may also exhibit different functions. Based on this, we have summarized in detail the functional differences between TLRs in the context of tumors. The last subsection is based on previous research on signaling pathways and specific tumor subtypes and provides a comprehensive overview of the latest treatment strategies for TLRs, especially clinical trial content.

Nonetheless, several challenges remain unaddressed in TLR research. (1) The scope of mechanistic research was relatively narrow. Most studies remain focus on specific TLR molecules in a particular cell type. For example, the TLR4 signaling pathway in macrophages has been extensively investigated. However, it remains unclear whether other TLRs in diverse cell lineages share the same pathways and whether the immune response initiated by TLRs is intricate and selective. (2) Despite sharing some intermediate signaling molecules and TFs, different TLRs can activate distinct immune responses. While chromatin remodeling offers some insights, the specific mechanisms involved demand deeper exploration. (3) Preclinical studies overwhelmingly rely on simplistic cell line‐derived xenograft models, which fail to simulate the true tumor immune response completely. Therefore, more transgenic mice should be used to construct spontaneous tumor model and TLR knock‐out or knock‐in mice, which are more convincing. (4) The field lacks comprehensive multiomics analyses, which can fully characterize the complete function of TLR in the context of tumors from the transcriptional to metabolic levels. It can also increase the tumor microenvironment feature map after the application of agonists and antagonists in vivo. (5) Although numerous TLR agonists and antagonists have shown promising effects in cell lines and animal models, their safety and efficacy in the human body still require further evaluation. Nanomaterials provide excellent therapeutic prospects, and clinical trials based on nanomaterials are increasing, with the hope of achieving safe and efficient delivery.

Despite these challenges, research based on TLR has achieved many transformative insights in the field of cancer. As tumor immunotherapy evolves into a cornerstone of modern oncology, TLR, as a key component of innate immunity, has undergone nearly 30 years of intensive research. From signaling pathway elucidation to tumor microenvironment modulation and therapeutic development, our understanding of TLR functions has deepened substantially. New treatment modalities are constantly emerging, and they have also played a critical role in clinical translation in various tumor patients. We anticipate that TLRs can achieve revolutionary results in tumor immunotherapy with increasing and deepening research.

## Author Contributions

Nueraili Maihemuti and Yueli Shi designed the study and drafted the manuscript and drew the figures. All authors participated in the revision of the manuscript. Kaiyue Zhang, Xinyuan Jiang, and Jiahe Chu participated in the design of the article, completed the table, and optimized the structure of manuscript. Kai Wang, Zhiyong Xu, and Yun Xu evaluated and reviewed the overall content, chapter logic, and language of the article. All the authors have read and approved the final manuscript.

## Ethics Statement

The authors have nothing to report.

## Conflicts of Interest

The authors declare no conflicts of interest.

## Data Availability

The authors have nothing to report.
